# ResNet and MaxEnt modeling for quality assessment of *Wolfiporia cocos* based on FT-NIR fingerprints

**DOI:** 10.3389/fpls.2022.996069

**Published:** 2022-11-02

**Authors:** YanYing Zhang, Tao Shen, ZhiTian Zuo, YuanZhong Wang

**Affiliations:** ^1^ Medicinal Plants Research Institute, Yunnan Academy of Agricultural Sciences, Kunming, China; ^2^ College of Traditional Chinese Medicine, Yunnan University of Chinese Medicine, Kunming, China; ^3^ College of Chemistry, Biology and Environment, Yuxi Normal University, Yuxi, China

**Keywords:** *Wolfiporia cocos*, fingerprints, residual convolutional neural network, quality assessment, maximum entropy model

## Abstract

As a fungus with both medicinal and edible value, *Wolfiporia cocos* (F. A. Wolf) Ryvarden & Gilb. has drawn more public attention. Chemical components’ content fluctuates in wild and cultivated *W. cocos*, whereas the accumulation ability of chemical components in different parts is different. In order to perform a quality assessment of *W. cocos*, we proposed a comprehensive method which was mainly realized by Fourier transform near-infrared (FT-NIR) spectroscopy and ultra-fast liquid chromatography (UFLC). A qualitative analysis means was built a residual convolutional neural network (ResNet) to recognize synchronous two-dimensional correlation spectroscopy (2DCOS) images. It can rapidly identify samples from wild and cultivated *W. cocos* in different parts. As a quantitative analysis method, UFLC was used to determine the contents of three triterpene acids in 547 samples. The results showed that a simultaneous qualitative and quantitative strategy could accurately evaluate the quality of *W. cocos*. The accuracy of ResNet models combined synchronous FT-NIR 2DCOS in identifying wild and cultivated *W. cocos* in different parts was as high as 100%. The contents of three triterpene acids in Poriae Cutis were higher than that in Poria, and the one with wild Poriae Cutis was the highest. In addition, the suitable habitat plays a crucial role in the quality of *W. cocos*. The maximum entropy (MaxEnt) model is a common method to predict the suitable habitat area for *W. cocos* under the current climate. Through the results, we found that suitable habitats were mostly situated in Yunnan Province of China, which accounted for approximately 49% of the total suitable habitat area of China. The research results not only pave the way for the rational planting in Yunnan Province of China and resource utilization of *W. cocos*, but also provide a basis for quality assessment of medicinal fungi.

## 1 Introduction

*Wolfiporia cocos* (F. A. Wolf) Ryvarden & Gilb., as a species of saprophytic medicinal fungi that can form edible sclerotia, is mainly distributed in many countries such as China, North America, and so on (Lee et al., 2012; Luo et al., 2020). Products based on *W. cocos* have been put into use in different fields, such as nutritional supplements, functional food, and cosmetics. With the increasing demand for *W. cocos*, the chemical components and pharmacological effects have become one of popular research directions. According to the literature reports, different parts of *W. cocos* have various types of applications when is put into clinical practice (Qian et al., 2018). Poria is the inner part of *W. cocos*, which is provided with a good effect on the treatment of kidney disease (Lee et al., 2012). The outer part, called Poriae Cutis, helps to treat edema and can also be used as a diuretic (Feng et al., 2013). *W. cocos* takes triterpene acid as the material basis to exert its pharmacodynamic effect, for example, dehydrotumulosic acid, dehydrotrametenolic acid, and poricoic acid A (Xia et al., 2014). Moreover, the accumulation ability of chemical components of wild and cultivated *W. cocos* is not the same, resulting in differences in content and changes in efficacy. Therefore, it is vital to find an accurate and effective modern technology to analyze wild and cultivated *W. cocos* in different parts.

Compared with DNA sequencing, which requires expensive experimental funds and rich knowledge, spectroscopy technology is known for its green, fast and simple operation (Pasquini, 2018). But the complexity of spectroscopy makes it difficult to interpret directly. Quantum chemical calculation is a powerful analytical means to solve the problem of spectral interpretation, which can provide information about molecular structure, energy level transition and other related information, making the application of this field rising (Ozaki et al., 2021). Meanwhile, the emergence of analytical methods such as chemometrics and machine learning has also made spectral interpretation simple. Near-infrared (NIR) spectroscopy pertains to a common vibrational spectroscopy technology. Fourier transform near-infrared (FT-NIR) spectroscopy has an edge in terms of non-destructive and high sensitivity, and has been widely applied to the research of quality control (Li et al., 2022b). Its detection range is 12500-4000 cm^-1^, and this region can present the information of O-H, N-H and C-H bonds vibration, chemical structure, and intermolecular or intramolecular interactions (Bec et al., 2021). However, signal peaks of the one-dimensional (1D) FT-NIR spectroscopy overlap seriously, making it difficult to extract feature information (Dong et al., 2021a). In 1993, Noda proposed Generalized two-dimensional correlation spectroscopy (2DCOS), which helps to highlight spectral features that are difficult to observe in 1D FT-NIR spectroscopy and enriches functional group information (Noda, 1993). 2DCOS can significantly improve the spectral resolution and reveal the correlation between frequencies, which is favorable to remedy the defects of conventional spectra (Karthikeyan et al., 2022). 2DCOS is to monitor 1D FT-NIR spectral signals under certain chemical or physical stimuli, and then convert them into synchronous 2DCOS images. While some differences in synchronous 2DCOS images can be directly observed, some small differences are difficult to be recognized by the naked eye. Therefore, it is necessary to use image recognition tool to make the purpose of identification come true.

In the past decade, convolutional neural network (CNN) has demonstrated its extremely mighty feature extraction ability and excellent classification performance, thus occupying a leading position in the domain of computer vision (Huertas-Tato et al., 2022). CNN can lessen the quantity of parameters and learning time through convolution kernels (Debus et al., 2021). However, as the network layers deepening, which lead to CNN model degraded rapidly. In contrast with the CNN model, the residual convolutional neural network (ResNet) can train a deep network with more than 1000 layers because the model can solve the problem of gradient disappearance by introducing shortcut connections (He et al., 2016; Ma & Mei, 2021). ResNet has good convergence and accuracy, so it is used as an effective tool for identification. ResNet18 and ResNet50 are used as the skeleton of the deep computer vision system (DCVS) to classify cocoa, the results showed that DCVS (ResNet18), with an accuracy rate of 96.82%, has the best classification effect compared with the CVS combined with traditional machine learning algorithm (Lopes et al., 2022). Yue et al. took *Paris polyphylla* as the research object and compared the identification performance of partial least squares-discriminant analysis, support vector machine, and ResNet (Yue et al., 2021a). The results showed that ResNet combined 2DCOS images with the strongest ability to identify medicinal plants. Liu et al. found that ResNet combined with 2DCOS images could be able to act as a robust model for tracing the geographic origin of *Amomum tsao-ko* Crevost et Lemaire, and the effect of synchronous 2DCOS images was better (Liu et al., 2021b). The results of two studies aforementioned illustrated the superiority of ResNet combined with synchronous 2DCOS images as a recognition method. In addition, ResNet combined with 2DCOS images has achieved remarkable results in the classification of *Eucommia ulmoides* (Li et al., 2022a), bolete mushrooms (Dong et al., 2021a) and other species. It has been reported that different parts of *W. cocos* could be authenticated successfully by 2DCOS combined with Resnet, but this study did not explore the effects of wild and cultivated on the quality of *W. cocos* (Li et al., 2022b). Using ResNet combined with 2DCOS to comprehensively evaluate the quality of wild and cultivated *W. cocos* in different parts is one of the focuses of this study.

The contents of chemical components of medicinal fungi depend partly on the suitable habitat (Li et al., 2020). The complex terrain of Yunnan and a wide variety of climate types due to its special geographical location provide favorable conditions for the growth and development of medicinal fungi (Yue et al., 2021b). So far, there are relatively few studies on the growth and distribution of *W. cocos*. In order to further evaluate the quality of *W. cocos*, it is essential to predict the distribution of its suitable habitat in Yunnan through the maximum entropy (MaxEnt) model. MaxEnt is thought as a mature method to provide a solution for the distribution and structure optimization of medicinal fungi (Yang et al., 2022).

In this study, 839 FT-NIR spectra were converted into synchronous 2DCOS images, and ResNet was used to analyze images to achieve the purpose of identifying samples from wild and cultivated *W. cocos* in different parts. Although infrared spectroscopy has been increasingly used to characterize the overall chemical information of herbal medicines, it is undeniable that liquid chromatography is still the mainstream method to detect chemical component content. Therefore, the differences of chemical component content of different *W. cocos* samples were analyzed by ultra-fast liquid chromatography (UFLC). Both spectroscopic and chromatographic methods were used for analysis, which complement each other and improve the reliability of the research results. Based on qualitative and quantitative analysis, it is planned to conduct comprehensive quality assessment on wild and cultivated *W. cocos* in different parts. Furthermore, the MaxEnt model as a powerful tool to predict suitable habitats for *W. cocos* which are distributed in China. We hope that the study can lay a foundation for the resource utilization and rational planting of *W. cocos*, and provide an effective method that can be widely applied to the quality assessment of medicinal fungi.

## 2 Materials and methods

### 2.1 Samples collection and pretreatment

*W. cocos* samples included 512 Poria (347 wild samples and 165 cultivated samples) and 327 Poriae Cutis (175 wild samples and 152 cultivated samples) from Yunnan, Anhui Province of China, were collected for subsequent spectroscopic analysis. The Poria (117 wild samples and 151 cultivated samples) and Poriae Cutis (128 wild samples and 151 cultivated samples) from 31 sampling sites were randomly selected for chromatographic analysis. Since the integrity of some samples was destroyed during the collection and transportation process, there were only Poria or Poriae Cutis in some sampling sites. Some samples in sampling sites were shown in **Figure S1**. And sampling sites distribution of *W. cocos* was displayed on **Figure S2**. After collecting samples, using a soft brush to clean the soil and wood debris covering the fresh samples. Next, place all samples in the shade for open-air drying. Until the water has almost evaporated in the samples, put them in an oven for further drying to constant weight at 50°C. Afterwards, each sclerotium of *W. cocos* was divided into Poria and Poriae Cutis, which were fully ground and the powder with homogeneous size was screened through a 60-mesh stainless steel sieve. At last, the powder of *W. cocos* samples was put into polyethylene zipper bags and stored in cool and dry conditions for further analysis.

### 2.2 FT-NIR spectra acquisition

*W. cocos* samples’ spectra were collected by FT-NIR spectrometer (Thermo Scientific Inc., Antaris II). This instrument needed to be warmed up for 2 hours before collecting samples’ spectra to ensure its stability and accuracy. A sufficient amount of sample powder was placed in the sample cup. Under the condition of the spectral resolution of 8 cm^-1^ with a scanning range of 10000-4000 cm^-1^, the scanning times of each sample were 64, and the samples were collected twice in parallel. In this study, the average spectrum of the sample was used as the data source for subsequent NIR spectrum modeling. It is worth mentioning that the background signal needs to be collected every hour for correction to eliminate the interference of air. In addition, when collecting spectral information, it is necessary to make the air humidity and temperature stay at 45°C and at 25%, respectively.

### 2.3 UFLC analysis

#### 2.3.1 Reagents and standards

We purchased purified water from Hangzhou Wahaha Group Co., Ltd. (Hangzhou, China). Chromatographic mobile phases A and B were supplied by Dikma Technologies (Lake Forest, CA, USA) and Thermo Fisher Scientific (Fair Lawn, NJ, USA), respectively. With the exception of the above two reagents, the remaining chemicals and reagents all belong to analytical grade. The standard references of poricoic acid A and dehydrotrametenolic acid with 98% purity were obtained from Beijing Keliang Technology Co., Ltd (Beijing, China). The standard references of dehydrotumulosic acid with 96% purity was provided by ANPEL Laboratory Technologies Inc. (Shanghai, China).

#### 2.3.2 Chromatographic conditions

UFLC fingerprint analysis were performed on a Shimadzu UFLC system. All samples shall be treated as follows before injection: Add 2 mL methanol into sample powder of 0.5000 ± 0.0001 g accurately weighed, and then ultrasonic extraction for 40min. A filter membrane with 0.22 μm micropore was used to filter the extract. The chromatographic separation of the extract was performed on an Inertsil ODS-HL HP column (3 μm, 3.0 × 150 mm) by using the conditions of mobile phases were (A) 0.05% formic acid in water and (B) acetonitrile, and the elution current velocity was 0.4 mL min^-1^. The gradient elution procedure for this experiment was as follows (Wang Q. Q. et al., 2020): (a) 0-25 min, B 40%; (b) 25-52 min, B 40-69%; (c) 52-56 min, B 69-72%; (d) 56-58 min, B 72-78%; (e) 58-58.01 min, B 78-90%; (f) 58.01-60 min, B remaining at 90%. The maximum absorption wavelength of 242 nm, of three triterpene acids was set as the detection wavelength. 7 μL was the injection volume in this study, and the chromatography column temperature was set to 40°C.

#### 2.3.3 2DCOS image acquisition

Discrete generalized 2DCOS algorithm is a powerful tool for spectral data analysis, and thus was utilized to generate the two-dimensional correlation spectrum at the present study. Due to the influence of external disturbances, a series of spectra can be captured in the generalized 2DCOS. With the help of cross-correlation analysis, the spectral intensity changes caused by disturbances can be generated into synchronous 2DCOS, which significantly improves the resolution (Noda, 2018). Its dynamic spectral intensity is expressed as *S*, where *v* and *t* represent variable and external disturbance, respectively (Noda, 2018):


(1) $$S\left(v\right)=\left\{\begin{array}{c} s\left(v,\;t_{1}\right)\\ s\left(v,t_{2}\right)\\ s\left(v,t_{3}\right)\\ .\\ .\\ .\\ s\left(v,t_{m}\right) \end{array}\right\}$$

Dong et al. found that ResNet combined synchronous 2DCOS images had good performance for identifying fungi, so this study only discussed synchronous 2DCOS (Dong et al., 2021b). The synchronous two-dimensional correlation intensity (Φ) of *v*_1_ and *v*_2_ at different frequencies is calculated by the following expression:


(2) $$\text{Φ}\left(v_{1},v_{2}\right)=\frac{1}{m-1}S{\left(v_{1}\right)}^{T}\cdot S\left(v_{2}\right)$$

In this study, divide the modeling data (90%) into training set (60%) and test set (30%) by using Kennard-Stone algorithm. The external validation set made up the remaining 10%. Finally, synchronous 2DCOS images automatically drawn by the computer program (matlab 2017a) were saved as JPEG image format which would be employed to subsequent modeling analysis.

#### 2.3.4 ResNet

The architecture of deep learning is a multilayers stack that consists of simple modules, which is an effective way for analyzing and processing large amounts of data. CNN is judged as a popular deep learning model, which is capable of extracting features of input data automatically based on forward and backward propagation principle (Liu et al., 2021a). Due to the advantage of learning complex non-linear features of images, CNN is generally utilized in image processing and image classification (McNeely-White et al., 2020). When the two-dimensional image P (x, y) is used as the input data of this model, a new image P’ (x, y) can be generated with the help of convolution kernel *f* (*s*, *t*). The mathematical representation is defined as Equation (3) (Dong et al., 2020):


(3) $$\text{P’}(\text{x,y})=\sum_{s}\sum_{t}f(s,t)\times P(x-s,y-t)$$

However, the CNN model has a fatal defect, that is, the gradient will disappear as the network deepens. ResNet was proposed to address this phenomenon through shortcut connection (He et al., 2016). In this study, the established model included two kinds of shortcut connections. One is the convolution block used when the input dimensions are inconsistent with output. Another is identity block, which is used when the situation is completely opposite to the convolution block. Compared with CNN, ResNet is apt to train and optimize, the accuracy can be improved with the increase of depth. Moreover, simplified output to an objective function that is *F*(*x*) = *H*(*x*)-*x*, which meant only the residual between input and output needs to be learned in this model, which greatly reduced the difficulty of learning (Dong et al., 2021a). In this study, ResNet model was applied to process two-dimensional images directly with the help of weight sharing and convolution operation, which contribute to improve the efficiency of training and build a robust model (LeCun et al., 2015; Dong et al., 2021b). A 12-layer ResNet was built, and the structure of which was shown in **Figure S3**. It mainly consists of five layers: convolutional, activation, pooling, fully-connected, and softmax layer. The convolution layer performs a convolution operation between the input data matrix and filters to generate a feature map. The output data of previous layer can be realized non-linear activation by the rectified linear unit (ReLU) function at activation layer in this study. Pooling layer, namely down-sampling layer, has the ability to cut down the amount of parameters and the spatial dimensions of feature maps to prevent overfitting. The proposed model made use of fully-connected layer to sort out the input two-dimension images through integrating feature information. Finally, softmax layer carries out the output in the form of probability. In addition to the above five layers, the introduction of BatchNorm layer aims to normalize the input data and accelerate the training speed of the model. What’s more, Stochastic Gradient Descent (SGD) not only determined the optimal parameters, but also minimized the cross-entropy loss function value, which is conducive to obtain the optimal model. And two convolution residual blocks and three identity blocks were used to extract the features of processed data. The ResNet identification strategy of wild and cultivated *W. cocos* in different parts was displayed in **Figure S4**.

### 2.4 The distribution of suitable habitat for *W. cocos*

#### 2.4.1 Species occurrence data

There were two ways to obtain the occurrence records of *W. cocos*. One was field survey. From 2012 to 2018, our research group investigated 91 distribution areas of *W. cocos*, and obtained detailed information including longitude, latitude and altitude through handheld GPS. Another was derived from literature review and government news reports, which in combination with Google satellite was used to investigate and analyze images for intentional cultivation regions of *W. cocos* form 2020-2022. In order to avoid sampling deviation, rarefied occurrence records by using ArcGIS software 10.0 (Esri, Redlands, California USA) at a 1 km^2^ spatial resolution. And then, we filtered and sorted out the species distribution data. Using Google Earth to determine occurrence records with ambiguous geo-coordinates, which would be deleted because of without detailed information or duplicate. Finally, a total of 198 occurrence records were obtained, which were stored in CSV format for subsequent modeling analysis.

#### 2.4.2 Environmental variables

*W. cocos* is an annual fungus, which requires temperature, humidity, solar radiation, etc. for its growth and development (Wang Q. et al., 2020). Therefore, we selected in the aggregate to 90 environmental variables for modeling, which have a bearing on the distribution of *W. cocos* in present study. Details of environment variables were shown in **Table S1**. Both elevation data and climate variables were derived from the WorldClim-Global Climate Data (https://www.worldclim.org/) with spatial resolution of 30′′. In National Tibetan Plateau Data Center (https://data.tpdc.ac.cn/zh-hans/), we acquired soil variables. Vegetation type variables and ≥10°C active accumulated temperature were obtained by Chinese Academy of Sciences (RESDC) (http://www.resdc.cn).

Strong collinearity among environmental variables can lead to overfitting of MaxEnt models in high-dimensional spaces (Peterson & Nakazawa, 2007). To address this issue, Spearman correlation analysis was employed to eliminate highly correlated environmental variables. If the coefficient is greater than 0.8, the correlation between variables is considered to be strong, so variables with less ecological significance were eliminated (Shen et al., 2021). **Table S2** showed the final environmental variables related to establish MaxEnt model for *W. cocos*, they were precipitation from August to November (Prec 08-11), mean diurnal range (Bio 02), min temperature of coldest month (Bio 06), max temperature of warmest month (Bio 05), temperature annual range (Bio 07), min temperature in January (Tmin 01), elevation (Ele), topsoil clay fraction (T_Clay), topsoil organic carbon (T_OC), topsoil silt fraction (T_Silt), topsoil sand fraction (T_Sand), and topsoil pH (H_2_O) (T_pH_ H_2_O). It should be noted that the environment variables used for modeling all need to be converted into ASCII format to form the environment layer.

#### 2.4.3 Establishment and evaluation of MaxEnt model

The MaxEnt model, as a means of popular species distribution model, can take into account the influence of climatic and non-climatic factors (topography, soil and vegetation, etc.) on the distribution of species (Shi et al., 2021; Yang et al., 2022). The growth and development of *W. cocos* has strict requirements on soil and vegetation type, which were defined as limiting factors in present study (Guo et al., 2019; Wang Q. et al., 2020). Therefore, two models were constructed to predict the distribution of *W. cocos*, one based on climatic factors and the other based on limiting factors (Guo et al., 2019). The overall suitable habitat distribution map of *W. cocos* was obtained by overlapping the distribution map of climate suitable habitat with the non-climate suitable habitat.

70% of the total data were used as training set to train the species distribution model of *W. cocos*. The remaining 30% were not involved in modeling and were selected as test set for evaluating the model performance. In present study, the modeling was repeated 30 times to reduce uncertainty and contingency. The iterations of this model were set to 5000. And the robustness of the model prediction was appraised by the area under the curve (AUC) of the receiver operating characteristics (ROC). The criteria for evaluating model performance according to AUC value were as follows: (a) 0.9-1.0, best; (b) 0.8-0.9, fine; (c) 0.7-0.8, average; (d) 0.6-0.7, reasonable; (e) 0.5-0.6, poor (Swets, 1988).

There are many environmental factors affecting the growth and development of *W. cocos*. In order to further clarify the importance of various environmental variables to this species, the main factors were determined by the Jackknife and the cumulative contribution rate of environmental variables (Shen et al., 2021). When environmental variables with cumulative contribution rate greater than 85%, which were considered as the main factors. And the results of Jackknife test presented the importance of environmental variables. Besides, the relevance between the distribution of *W. cocos* and the environmental variables used for modeling were emerged from response curves.

In the end, converted the MaxEnt prediction results in ASCII format to raster data through the conversion tool in ArcGIS. The range of the suitability index obtained from the results of the MaxEnt model was 0 to 1, and the threshold value of 0.5 was used as the basis for dividing the suitability. Therefore, the habitat suitability of *W. cocos* was divided into two types: suitable (suitability index ≥ 0.5) and unsuitable (suitability index < 0.5) (Guo et al., 2019). Through field visit and related literatures, we found that *W. cocos* can only grow in a specific vegetation type, that is, coniferous and broad-leaved mixed forest dominated by conifers with a few broad-leaved trees (Wang Q. et al., 2020). Therefore, coniferous forest, mixed coniferous forest and broad-leaved forest were considered as suitable vegetation types, and the remaining vegetation types were unsuitable.

## 3 Results and discussion

### 3.1 Interpretation of FT-NIR spectrum

**Figure 1:**
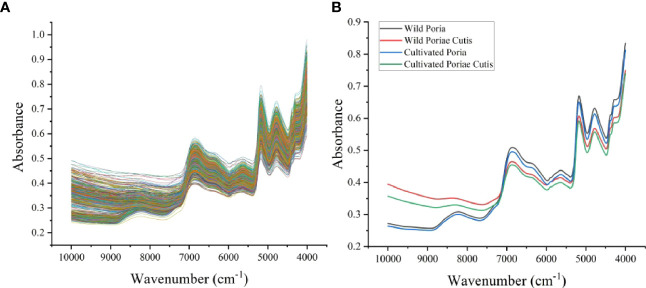
Spectra of samples: **(A)** raw spectra, **(B)** average spectra.

To highlight the differences between samples, a rough visual comparison of the FT-NIR spectra of wild and cultivated *W. cocos* in different parts was performed (**Figure 1A**). As a result of the highly similarity and severe overlap of different samples, we further analyzed the average spectra of wild and cultivated *W. cocos* in different parts (**Figure 1B**). The characteristic absorption peaks of the **Figure 1B** were analyzed as follows: the peak located between 8380 and 8230 cm^-1^ is associated with unsaturated lipids and represents the second overtone of C=C-H stretching (Schütz et al., 2022). Two intensive moisture-related absorption bands at approximately 6870 cm^-1^ and 5180 cm^-1^ exhibit the first overtone of O-H and polysaccharide combination band of O-H stretching vibration, respectively (Zhao et al., 2014). The absorption band arises from the first overtone of -C-H stretching at the range of 5730-5560 cm^-1^ (Alamprese et al., 2013). The combination of amide bond vibrations causes an absorption band in the range of 4985-4515 cm^-1^, which is corresponding to proteins (Büning-Pfaue, 2003). The peak within 4255 and 4320 cm^-1^ is assigned to the combination of carbon single bond stretching and single bond deformation (Long et al., 2022). NIR spectrum expresses the accumulation of the main components of samples in the form of absorption bands. As shown in **Figure 1**, the appearance and trend of spectral profiles of different samples were similar, but there were some slight differences in absorbance, reflecting the different accumulation of chemical components in different samples. However, the visual difference was too small to support the raw spectrum as the basis for identifying different samples directly. Therefore, it is need to take action to apply machine learning to further identify wild and cultivated *W. cocos* in different parts.

### 3.2 Data visualization

**Figure 2:**
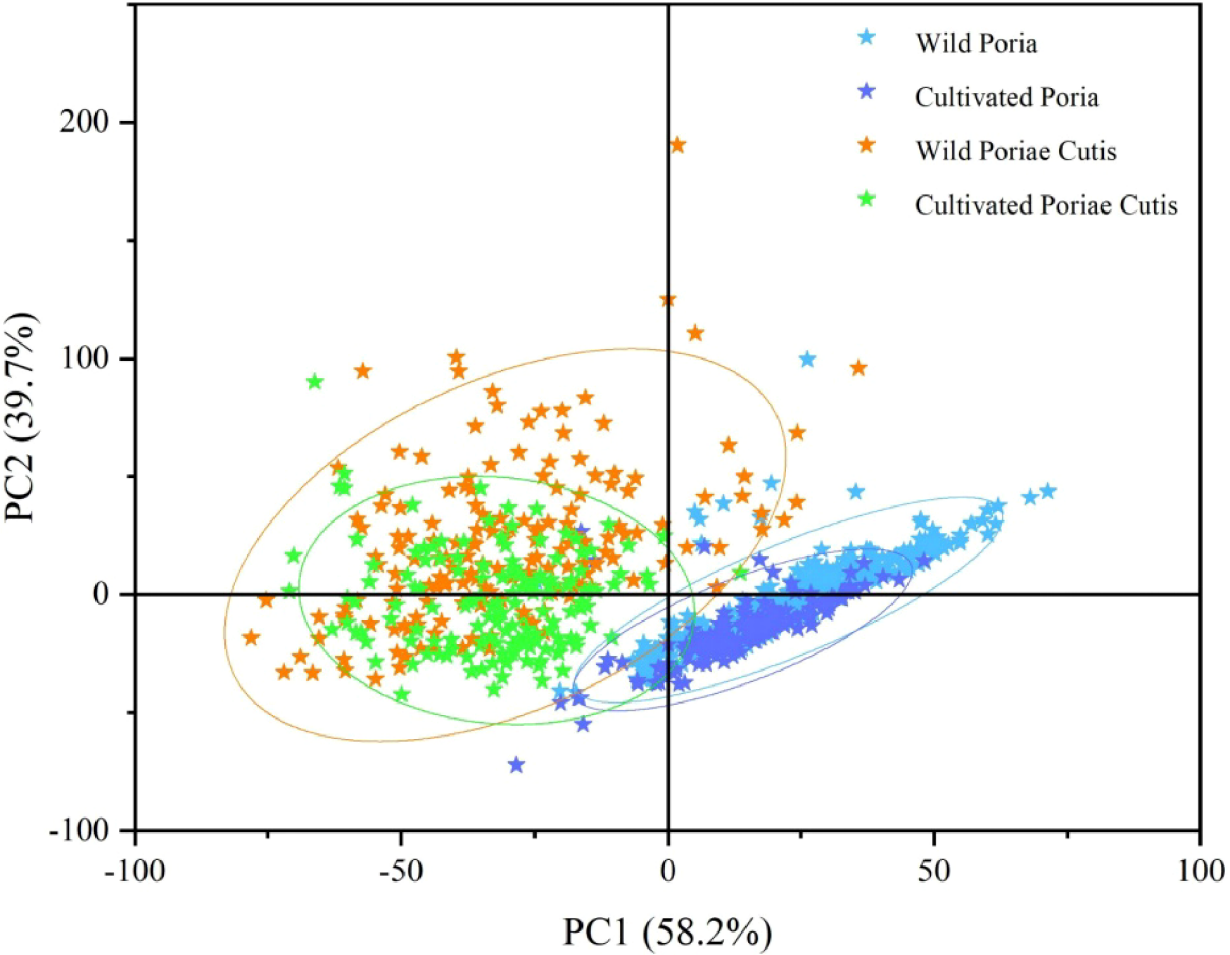
PCA score plot based on FT-NIR spectra.

As a tool for extracting feature variables and reducing dimensionality, principal component analysis (PCA) can transform a set of correlated original variables into non-correlated variables through linear transformation. In this study, PCA was used for the visualization of the FT-NIR spectral dataset, so as to present the similarity and difference of different samples in visual effect. **Figure 2** showed the PCA score plot for FT-NIR spectral data based on four classes (wild Poria, cultivated Poria, wild Poriae Cutis, and cultivated Poriae Cutis), with PC1 explained 58.2% of variance and PC2 explained 39.7%. It can be intuitively seen that two clusters were formed by the first two principal components in PCA score plot. It was speculated that the reason for the good separation effect of Poria and Poriae Cutis was that different parts have different accumulation capacities for chemical components, resulting in significant differences in chemical component or content. Unfortunately, the wild and cultivated samples from the same part cannot achieve good separation, and some samples even have severe overlap (in **Figure 2**). The reason for this phenomenon may be that the geographical environments of wild and cultivated samples in the same parts were different, which were mainly affected by the climate and origin. Due to the joint action of many factors, the separation of the four classes cannot be fully achieved by PCA alone, so ResNet was further used in this study to identify wild and cultivated samples from different parts.

### 3.3 2DCOS spectrum analysis and ResNet

**Figure 3:**
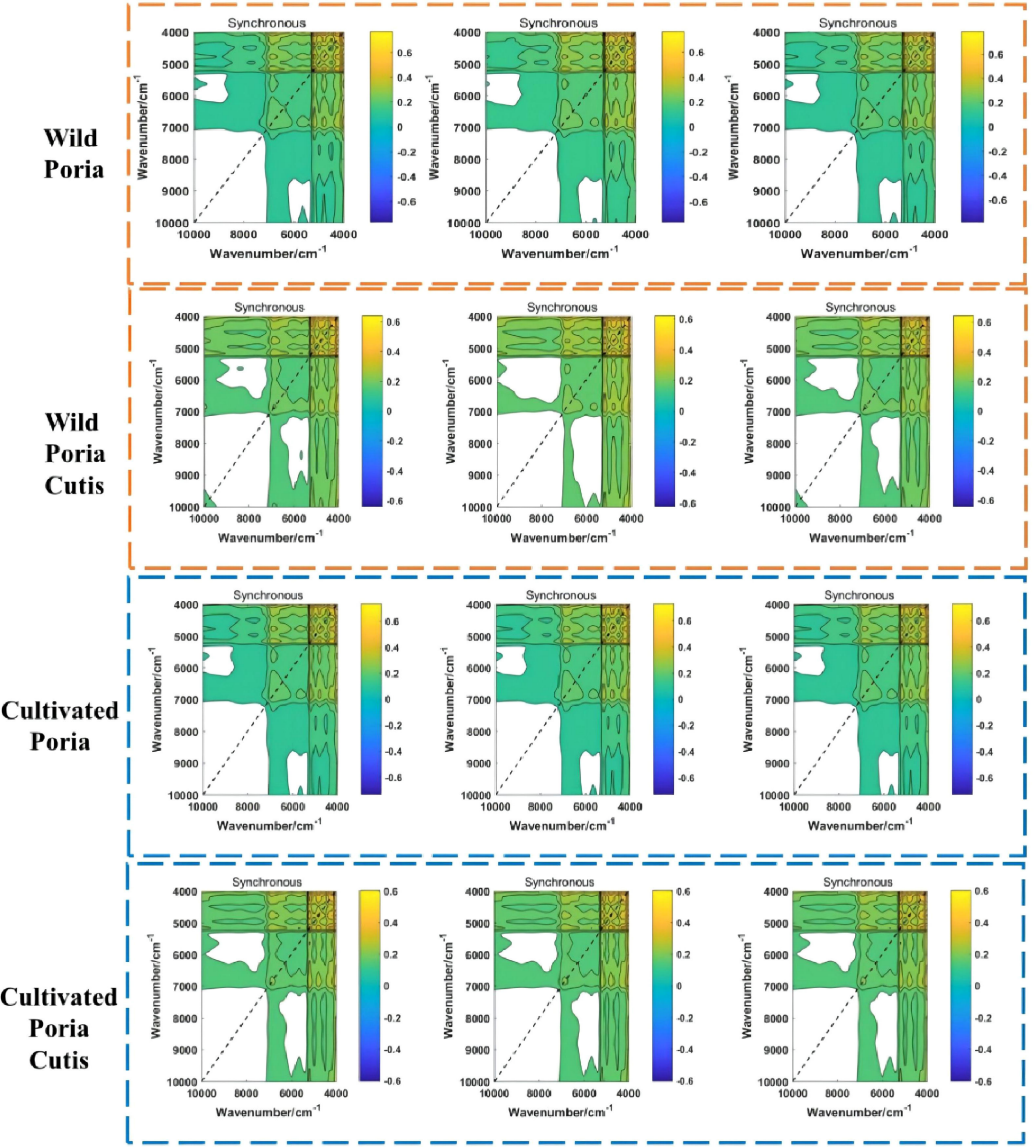
The synchronous 2DCOS images of wild and cultivated *W. cocos* in different parts.

In this study, synchronous 2DCOS images of wild Poria, cultivated Poria, wild Poriae Cutis, and cultivated Poriae Cutis altogether totaled 839 were drawn, which were displayed on **Figure 3**. The synchronous 2DCOS image’s axis of symmetry is diagonal, and the auto peaks located on the diagonal, which are generated by the autocorrelation results of the dynamic fluctuations caused by the perturbation (Huang et al., 2003). Auto peaks show the susceptibility of correlation spectral changes in different regions (Dong et al., 2021a). There are cross peaks on two flanks of the diagonal, which express the synchronization changes of spectral signals at different wavenumbers, reflecting the coupling effect between groups. Although the synchronous 2DCOS images have clear lines and rich colors, due to the subtle changes between some samples, they are almost indistinguishable with naked eyes. However, ResNet can directly analyze the image and enhance the identification ability. Therefore, it is necessary to identify different samples with the help of ResNet.

In order to identify synchronous 2DCOS images of wild and cultivated *W. cocos* in different parts, ResNet models were established with the weight attenuation coefficient λ and learning rate were 0.0001 and 0.01, respectively. The present study used the accuracy curve of training set and test set to assess the performance about identification of the established model. When the value is closer to 1 means its identification ability is stronger. The convergence effect of this model was expressed by the cross-entropy loss function. When the value is closer to 0 meant its convergence effect is better. Furthermore, the generalization ability of the established ResNet model was evaluated using external validation set.

**Figure 4:**
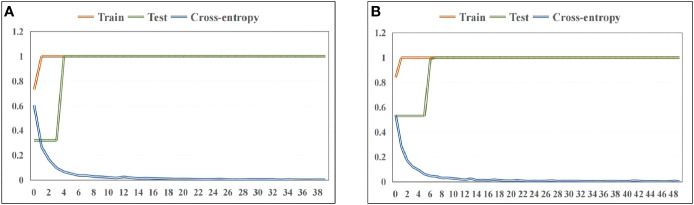
The accuracy curve and cross-entropy cost function of ResNet models based on wild and cultivated *W. cocos* in different parts: **(A)** Poria and **(B)** Poriae Cutis.

**Figure 4A** showed the accuracy curve and cross-entropy loss function of Poria based on synchronous 2DCOS spectra. It could be found from the figure that the accuracy of training set and test set was up to 100%. This result indicated that the ResNet model combined synchronous 2DCOS spectra had excellent identification ability for wild and cultivated Poria. At the same time, when epoch reached 39, the loss value was equal to 0.003, which was very close to 0. It showed that this model had excellent convergence effect. The accuracy curve and cross-entropy loss function of Poriae Cutis about synchronous 2DCOS were displayed in **Figure 4B**. Similarly, training set and test set had high accuracy that was 100%, indicating that this model was also suitable for using to identify Poria Cutis. Although the epoch increased, the loss value showed a downward trend. When epoch reached 49, the loss value was equal to 0.002, which meant that the convergence effect of this model was satisfactory. The classification accuracy of external validation set of Poria and Poriae Cutis were 100%, that is, both wild and cultivated samples of Poria and Poriae Cutis were correctly classified. The confusion matrix of the classification results was shown in **Figure S5**. These results showed that the ResNet models were established in this study had good generalization ability.

Compared with traditional identification methods that require rich experience and lack objectivity, the ResNet model proposed in this study combined with synchronous 2DCOS has better identification accuracy. In conclusion, the ResNet model combined synchronous 2DCOS images could be used as an effective means to identify wild Poria, cultivated Poria, wild Poriae Cutis, and cultivated Poriae Cutis.

### 3.4 Chromatographic fingerprint analysis

**Figure 5:**
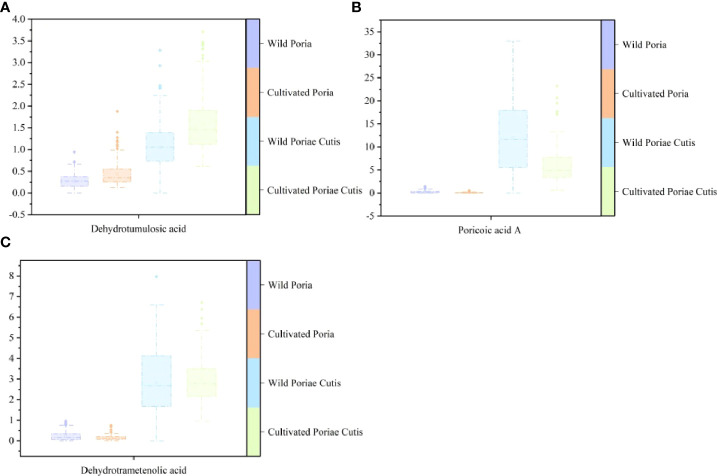
Box-plots based on contents of three triterpene acids of wild and cultivated *W. cocos* in different parts: **(A)** dehydrotumulosic acid, **(B)** poricoic acid A, and **(C)** dehydrotrametenolic acid.

The difference in absorbance in **Figure 1** may be caused by the difference of chemical components content in different samples. Liquid chromatography is a conventional method to detect the content of chemical components, and its fingerprint can provide comprehensive and overall chemical information. Therefore, UFLC was used to etermine three triterpene acids contents (dehydrotumulosic acid, poricoic acid A, dehydrotrametenolic acid) in randomly selected samples of Poria and Poriae Cutis. The box-plots of the contents of three triterpene acids were shown in **Figure 5**. It could be distinctly seen from the figure that wild Poriae Cutis had the highest content of poricoic acid A. The dehydrotumulosic acid content in cultivated Poriae Cutis was the highest, followed by wild Poria. And dehydrotrametenolic acid content in wild Poriae Cutis was highest than that in wild Poria, cultivated Poria, and cultivated Poriae Cutis. To make a long story short, the content of three triterpene acids in Poriae Cutis was significantly higher than that in Poria. And through research, it was found that the content of wild Poriae Cutis was more than that of cultivated Poriae Cutis.

Different parts of *W. cocos* have different accumulative abilities of chemical components, for the sake of exerting their medicinal effects with utmost extensions, they can be selected according to the needs during use. Therefore, the rational use of Poria and Poriae Cutis can relieve the pressure of resources. In particular, the premise of the generation and accumulation of chemical components is the suitable habitat required for the survival of species (Guo et al., 2021). As we know, the quality of medicinal fungi is decided by the content of chemical components. For reasons of obtaining high-quality *W. cocos*, it needed to predict its suitable habitat with the help of MaxEnt.

### 3.5 Analysis on the suitable habitat distribution of *W. cocos*

The ROC curves of MaxEnt models were showed in **Figure S6**. Form the resultant figure, the average AUC_test_ value of the MaxEnt model based on climate variables was 0.952 ± 0.008 (**Figure S6A**), and the MaxEnt model based on soil variables was 0.897 ± 0.015 (**Figure S6B**). The results demonstrated that the models proposed in this study has good performance and can accurately predict the distribution of *W. cocos*. Thus, the model can be used for subsequent analysis.

Using 17 environmental variables as indicators to simulate the suitable habitat distribution for *W. cocos*. According to the Jackknife test results and cumulative contribution rate of environment variables, the main factors obtained were as follows: (a) the main factor about climate were precipitation in August (Prec 08), precipitation in October (Prec 10), precipitation in November (Prec 11), Bio 02, and Bio 06 (**Figure S7A**); (b) the main factor about soil were T_Clay, T_OC, T_Silt, T_Sand, and T_pH_ H_2_O (**Figure S7B**); (c) the main factor about topography was Ele. Then, the suitable range of the above-mentioned main factors were obtained through the response curves (**Table 1**). The results demonstrated that the suitable range of Prec 08 was about 148~302 mm in MaxEnt model. The suitable range for Prec 10 was approximately 64~159 mm and Prec 11 was approximately 28~113 mm. The suitable range of Bio 02 and Bio 06 were approximately 7.51~11.47°C and -2.39~7.89°C, respectively. In the same way, the suitable range of the soil main factors and elevation were given in **Table 1**.

**Table 1:** The suitable range for main factors.

Environment variable	Units	Suitable range
Bio 02	°C	7.51~11.47
Bio 06	°C	-2.39~7.89
Prec 08	mm	148~302
Prec 10	mm	64~159
Prec 11	mm	28~113
Ele	m	132.4~2759.8
T_Clay	% weight	17.65~31.15, 31.22~61.60
T_OC	% weight	0.74~1.73
T_pH_H_2_O	/	2.94~5.47, 7.63~7.91
T_Sand	% weight	32.20~52.62, 17.07~32.08
T_Silt	% weight	16.17~37.50, 49.29~53.30

At present, there are few studies on the ecological characteristics of *W. cocos*. We have a preliminary understanding of the ecological characteristics of *W. cocos* by visiting surveys and reviewing existing literature reviews. Through investigation, we found that temperature is a significant factor for the growth and development of *W. cocos*, and its mycelium generally grows at a temperature of 15 to 35°C. It should be noted that the mycelium of *W. cocos* can withstand high temperatures above 40°C, but it will be dormant when the temperature is below 0°C (Jiang, 2021). Usually, the intentional cultivation time of *W. cocos* is from April to May. When the temperature range is 23~28°C and the relative air humidity is about 70%, the sclerotium grows very quickly and is conducive to the dispersal of spores. Although *W. cocos* is planted under the surface and does not require sunlight, it still needs to be cultivated in a sunny place, which can increase the diurnal temperature to promote the growth and development of *W. cocos* (Wang Q. et al., 2020). The sclerotia of the fungus begin to grow about two months after intentional cultivation, and the soil moisture content is very critical for its growth and needs to be maintained at about 25% (Jing et al., 2020). The distribution area of *W. cocos* should meet the above climatic conditions. The model concluded that the suitable range of climatic variables was consistent with the biological characteristics of *W. cocos*, explaining that the model results were reliable.

Furthermore, previous studies have shown that *W. cocos* grows mostly at altitudes ranging from 200-1000 m (Jiang, 2021). *W. cocos* distributed in Yunnan is mainly parasitic on the dominant tree species *Pinus yunnanensis* in this area (Zhang et al., 2022). *Pinus yunnanensis* mainly grows at an altitude of 700-3000 m (Wang et al., 2013). As a result, *W. cocos* is widely distributed in Yunnan and can grow at an altitude of more than 1000 m. The optimum soil pH for the growth of *W. cocos* is 4~6, which was obviously inconsistent with the results obtained by our model (Wang Q. et al., 2020). However, the data showed that the pH of soil in Yunnan is mostly between 5 and 6, indicating that it can meet the pH conditions for the growth of *W. cocos*. The soil type with loose ventilation, strong water permeability and more sand and less silt is favor of the growth of *W. cocos* (Cheng et al., 2021). Unfortunately, there are very few soil types that meet the above criteria. Our model results indicated that topsoil sand fraction was lower than the standard required for the growth of *W. cocos*. Therefore, the intentional cultivation of *W. cocos* requires modification of the soil type. And the suitable ranges of T_OC obtained by the model was in line with requirement for the growth and development of *W. cocos*. What’s more, due to *W. cocos* needs to be parasitized on different Pinus species to obtain nutrients required for growth and development, coniferous, mixed coniferous, and broad-leaved forests were selected as the suitable vegetation type in this study (Rios, 2011). To sum up, the distribution of suitable habitat of *W. cocos* is restricted by many factors, among which the vegetation type, soil, temperature and moisture have significant effects on the growth and development of *W. cocos*. Therefore, it is very important to select the appropriate vegetation type, modify soil type, and reasonably regulate temperature and moisture during the intentional cultivation of *W. cocos*. This is a prerequisite for the growth and development of *W. cocos*, laying the foundation for high quality and high yield of *W. cocos*.

**Figure 6:**
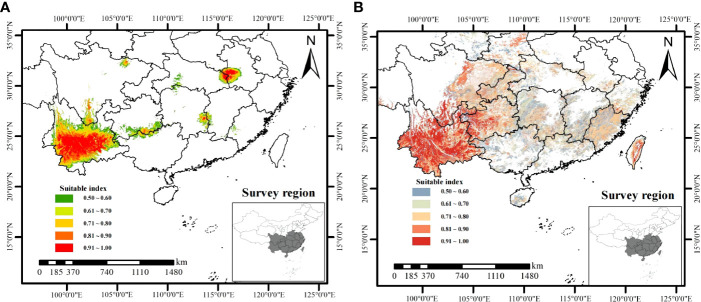
Prediction of **(A)** climate and **(B)** soil suitable habitat for *W. cocos*.

Using the combination of ArcGIS and MaxEnt to present the distribution of climate suitable habitat (**Figure 6A**) and soil suitable habitat (**Figure 6B**) for *W. cocos*. The climate suitable habitats of *W. cocos* were primarily distributed in Yunnan and the junction of Anhui and Hubei, and scattered in Sichuan, Guizhou, Guangxi, Fujian, Hu’nan and Jiangxi (**Figure 6A**). Suitable habitats about soil were widely distributed in the southwest, southeast and in central China (**Figure 6B**). The climate suitability distribution map and the soil suitability distribution map were overlapped to obtain the habitat suitability distribution map of *W. cocos* (**Figure S8A**). It is evident from the figure that most of the suitable habitats are located in Yunnan Province. According to the statistical analysis of the area of suitable habitat, which suitable for cultivating *W. cocos* in China is 6.89 × 10^4^ km^2^, and the area for suitable habitat in Yunnan accounted for approximately 49% of the total area.

As displayed in **Figure S8B**, the suitable habitat of *W. cocos* is distributed in most areas of Yunnan, and the specific information was shown in **Table 2**. The suitable habitat of Pu’er is the most widely distributed, with an area of 0.77 × 10^4^ km^2^. Followed by Chuxiong, the area of suitable habitat is 0.47 × 10^4^ km^2^. Dali ranks third with the area of habitat suitable of 0.36 × 10^4^ km^2^. The suitable habitat areas of Kunming, Honghe and Yuxi are very close to those of Dali, which are 0.34 × 10^4^ km^2^, 0.33 × 10^4^ km^2^ and 0.31 × 10^4^ km^2^, respectively. The suitable areas for the above-mentioned cities accounted for approximately 76% of the total suitable area in Yunnan. Most of the suitable habitats distributed in Yunnan are in the south, and a small part are in the west. The hydrothermal resources in southern Yunnan are better than those in eastern and western Yunnan, which are more conducive to the growth and development of *W. cocos*. Western Yunnan is affected by Hengduan Mountains, so the terrain fluctuates greatly and the temperature difference is large, which may be an important reason for the less distribution of suitable habitats.

**Table 2:** The distribution area of suitable habitat for *W. cocos*.

Province in China	Area (×10^4^ cm^2^)	City in Yunnan	Area (×10^4^ cm^2^)
Yunnan	3.38	Pu’er	0.77
Sichuan	0.75	Chuxiong	0.47
Hunan	0.37	Dali	0.36
Guizhou	0.28	Kunming	0.34
Anhui	0.27	Honghe	0.33
Jiangxi	0.16	Yuxi	0.31
Fujian	0.12	Qujing	0.25
Other	1.56	Other	0.55
Total	6.89	Total	3.38

In summary, the MaxEnt model can be used to predict the suitable habitat of *W. cocos*. The results illustrated that the suitable habitat of *W. cocos* is the most widely distributed in Yunnan, which can be used as the main production area of this species. Through the above modeling results, we can have a basic understanding of the suitable habitat distribution of *W. cocos* in Yunnan, and provide a reference for planting reasonably of this species in the later stage. At the same time, it is necessary to introduce advanced cultivation techniques to improve the yield of *W. cocos*.

### 3.6 Limitations and uncertainties

This study can further improve the evaluation of the suitable habitat for *W. cocos*, which is mainly given expression in the following four aspects: 1) The suitable habitat of *W. cocos* is the result of multiple factors, but this study only considers the influence of climate, soil and vegetation, ignoring the influence of human activities, which can be carried out in the follow-up research. 2) A limited number of occurrence records for this species were collected, so the sample size could be expanded in further study to achieve better model performance. 3) Due to the lack of precision of soil data, the accuracy of the model results has been affected to a certain extent. 4) In the follow-up research, the correlation between the content of chemical components and suitable habitats needs to be studied to determine the effect of suitable habitats on chemical components’ content in *W. cocos*. Despite the MaxEnt model proposed in this study has shortcomings, it was successfully utilized to study the distribution of suitable habitat of *W. cocos* in Yunnan for the first time.

## 4 Conclusions

The content of chemical components of medicinal fungi is easily affected by environmental factors, resulting in great differences in the quality of different samples. In this study, a scientific strategy based on FT-NIR spectroscopy and UFLC was developed to comprehensively evaluate the quality of *W. cocos*. The accuracy of the combination of synchronous 2DCOS images and ResNet models was as high as 100%, so as to realize the purpose of rapidly identifying wild and cultivated *W. cocos* in different parts. Then, UFLC was used to quantify the content of three triterpene acids in different *W. cocos* samples. The results indicated that the quality of wild Poriae Cutis was the best. Combined with qualitative and quantitative analysis is helpful to reveal the overall chemical profile of *W. cocos* and scientifically carry out a quality assessment for it. The suitable habitat has a significant stake in the accumulation of chemical components of medicinal fungi. For the sake of further analyzing the effect of environmental factors on suitable habitats’ distribution of *W. cocos*, a MaxEnt model was established. Under current climate, the suitable habitat of *W. cocos* is mainly distributed in Yunnan Province of China, which occupies about 49% of the total area. Yunnan has great development potential as the main planting area of high-quality *W. cocos*. However, further investigation is still needed to explore the relationship between quality and suitable habitat of *W. cocos*. The present study not only can provide a basis for improving the efficiency of resource utilization and rational planting of *W. cocos*, but also can provide a reference for the quality assessment of medicinal fungi.

## Data availability statement

The original contributions presented in the study are included in the article/**Supplementary Material**. Further inquiries can be directed to the corresponding authors.

## Author contributions

YZ, conceptualization, methodology, validation, data curation, and writing-original draft. TS, software, formal analysis, visualization, writing-review and editing. ZZ, investigation, supervision, and resources. YW, resources, project administration, and funding acquisition. All authors contributed to the article and approved the submitted version.

## Funding

This study was supported by National Natural Science Foundation of China (Grant number: 31860584), Special Program for the Major Science and Technology Projects of Yunnan Province (Grant number: 202102AA100010), and Special Program for the Major Science and Technology Projects of Yunnan Province (Grant number: 202202AE090001).

## Acknowledgments

I'm grateful for the valuable comments from editors and reviewers.

## Conflict of interest

The authors declare that the research was conducted in the absence of any commercial or financial relationships that could be construed as a potential conflict of interest.

## Publisher’s note

All claims expressed in this article are solely those of the authors and do not necessarily represent those of their affiliated organizations, or those of the publisher, the editors and the reviewers. Any product that may be evaluated in this article, or claim that may be made by its manufacturer, is not guaranteed or endorsed by the publisher.
