# Supplementary material for: ResNet and MaxEnt modeling for quality assessment of Wolfiporia cocos based on FT-NIR fingerprints
Source: Front Plant Sci. 2022 Nov 2;13:996069. doi: 10.3389/fpls.2022.996069 (PMC9666765; doi:10.3389/fpls.2022.996069)
Supplement: Supplementary file 1 [file DataSheet_1.docx]

Supplementary Material

Figure S1. Sample pictures of the sampling site, Poria and Poriae Cutis.

Figure S2. The distribution of sampling sites for *W. cocos*.

Figure S3. Framework of the established ResNet model.

Figure S4. ResNet identification strategy for wild and cultivated *W. cocos* in different parts.

Figure S5. The confusion matrix of ResNet model based on synchronous 2DCOS spectra: (A) Poria and (B) Poriae Cutis.

Figure S6. ROC curves of MaxEnt models for *W. cocos*: (A) climate and (B) soil.

Figure S7. The results of the jackknife test and the environment variables’ percent contributions: (A) climate variables and (B) soil variables.

Figure S8. The suitable habitat of *W. cocos* distributed in (A) China and (B) Yunnan Province.


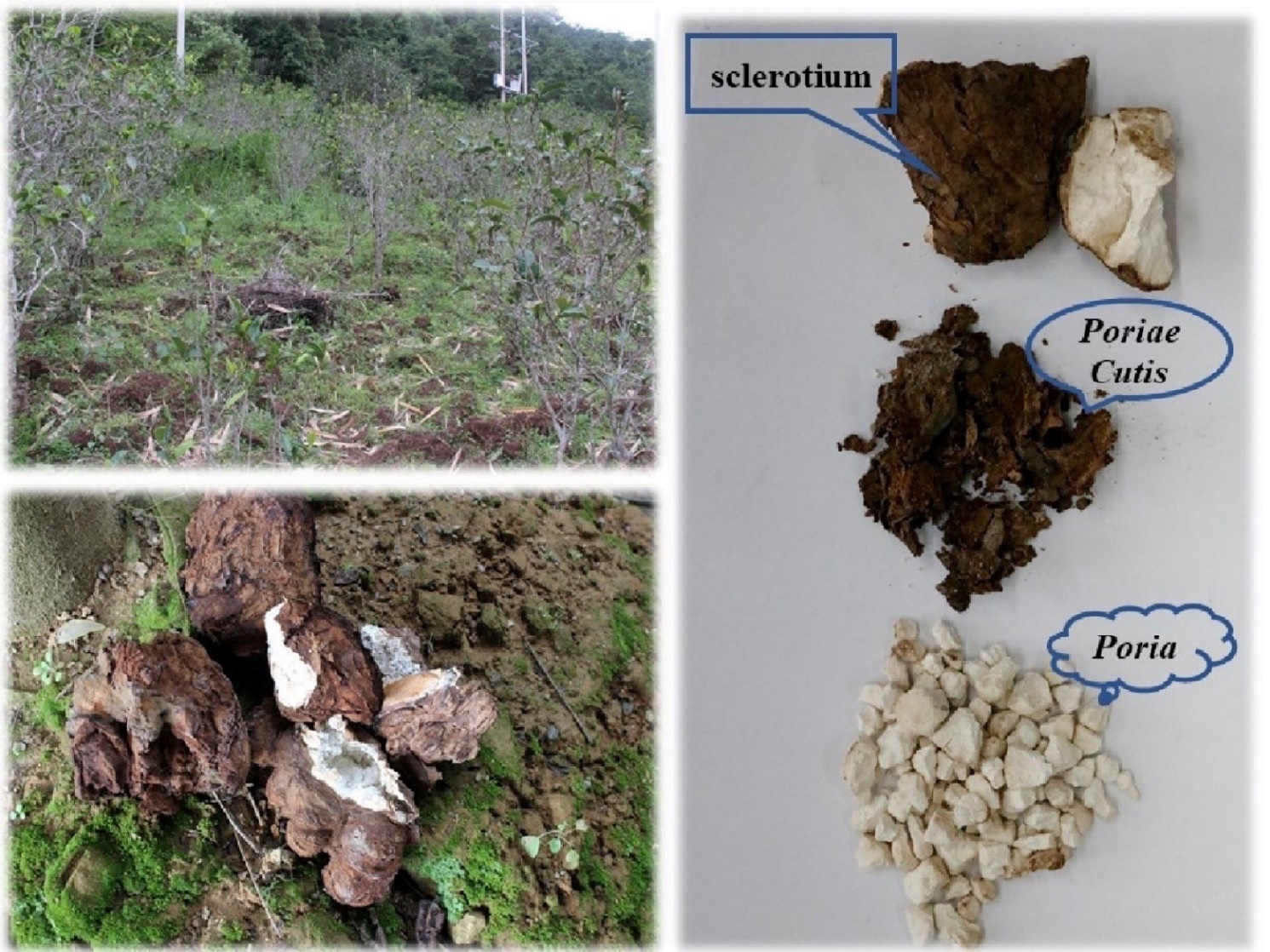


Figure S1. Sample pictures of the sampling site, Poria and Poriae Cutis.


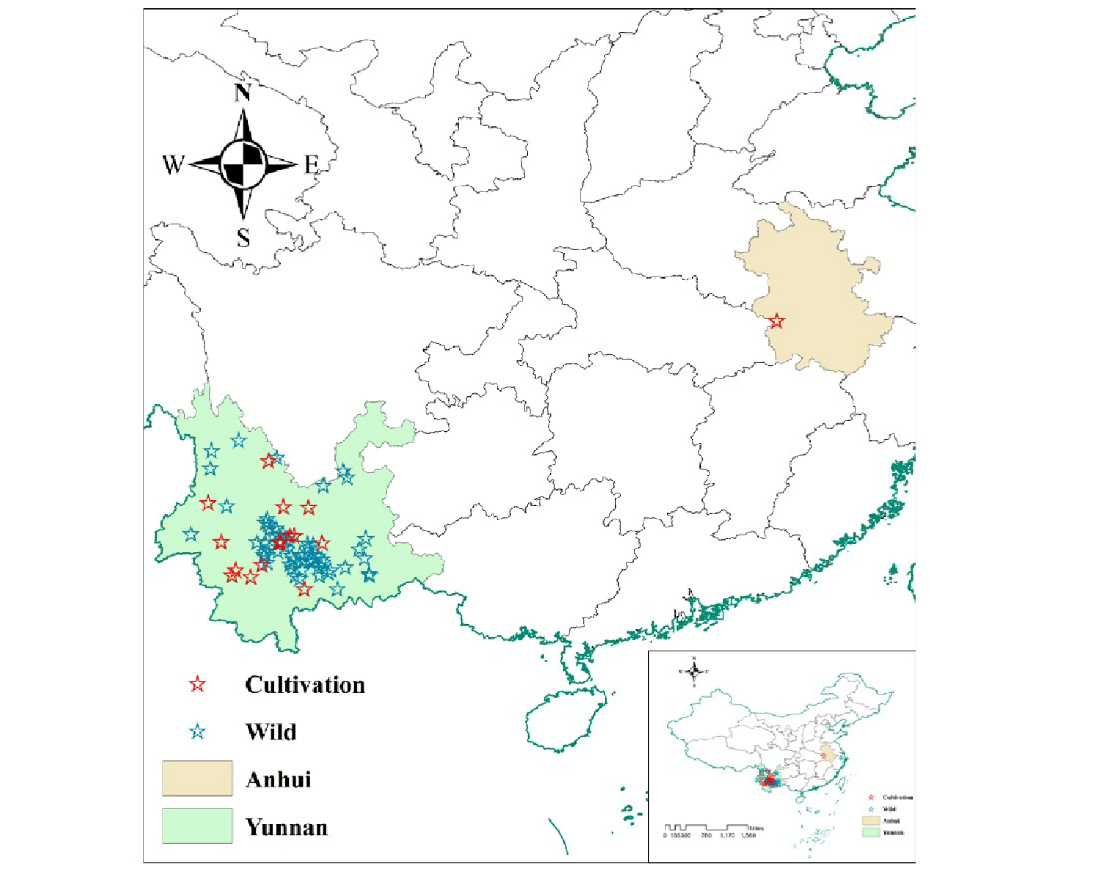


Figure S2. The distribution of sampling sites for *W. cocos*.


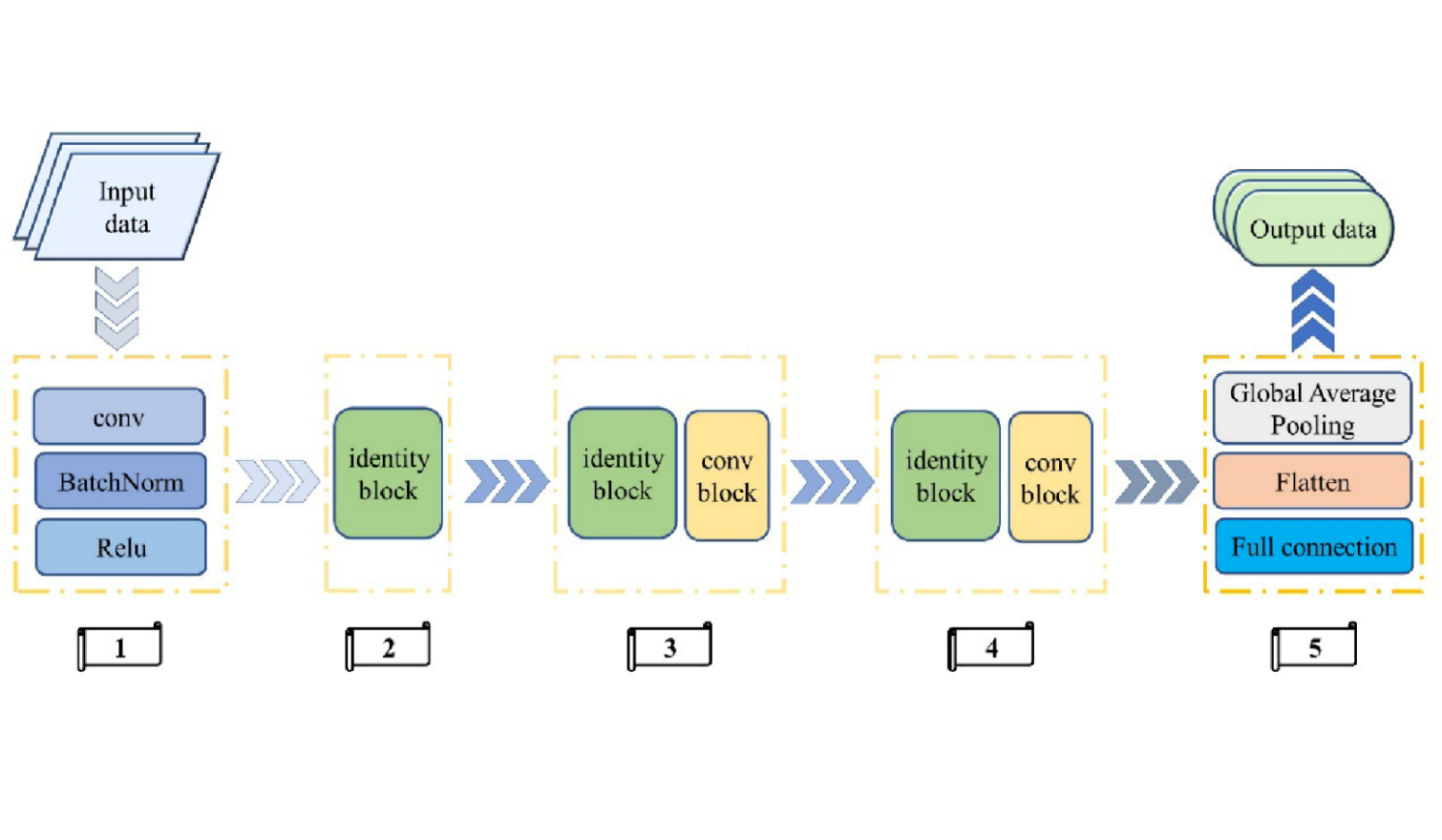


Figure S3. Framework of the established ResNet model.


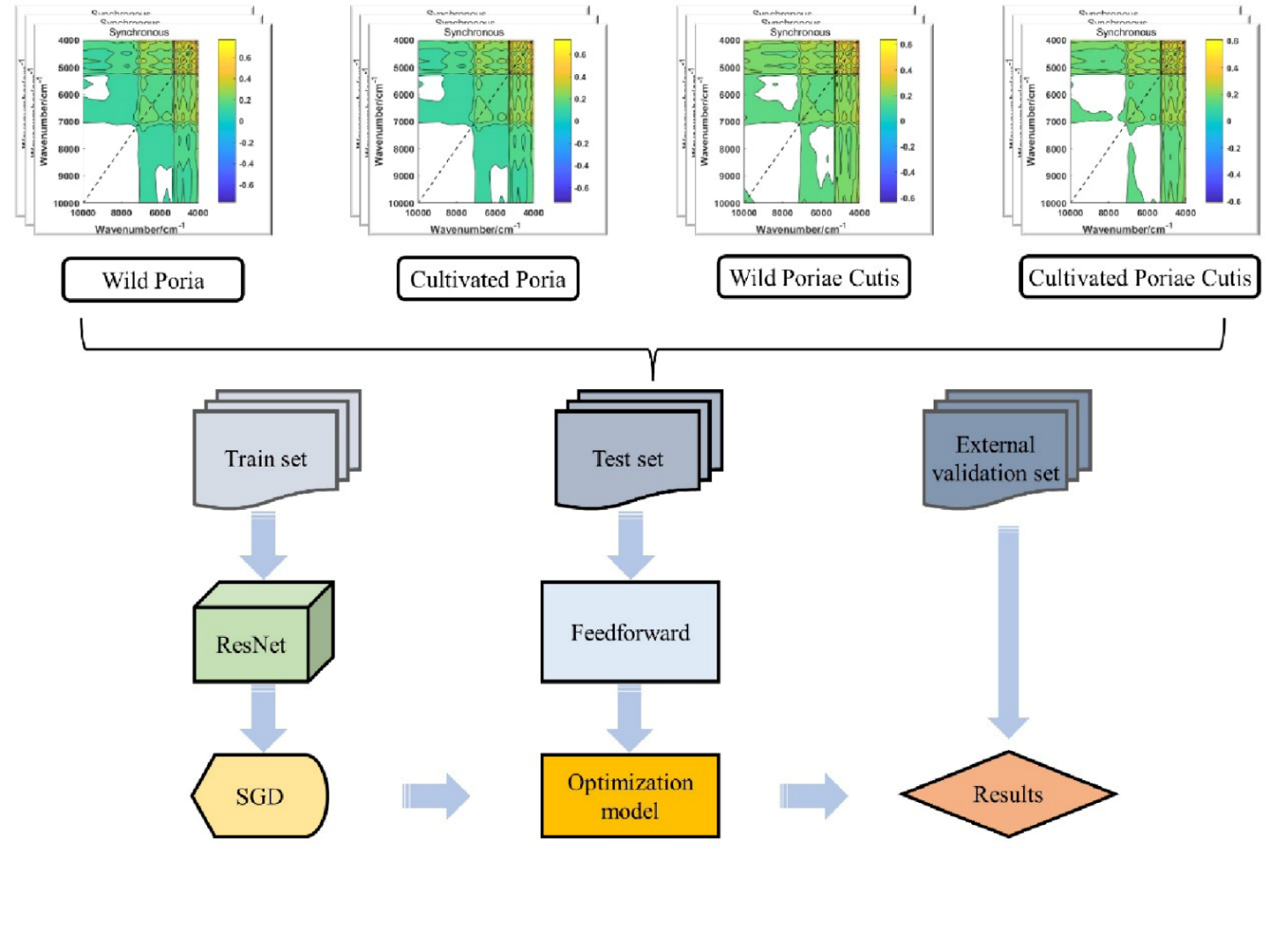


Figure S4. ResNet identification strategy for wild and cultivated *W. cocos* in different parts.


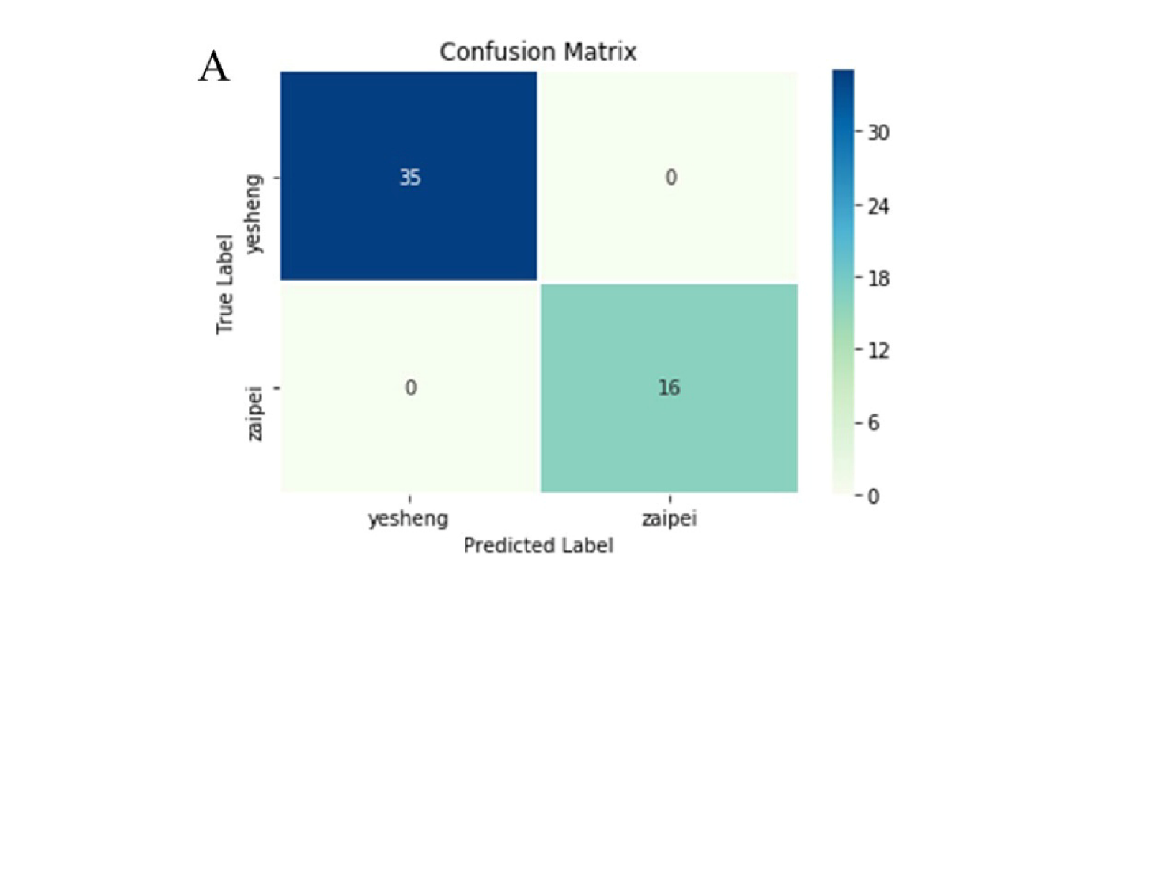

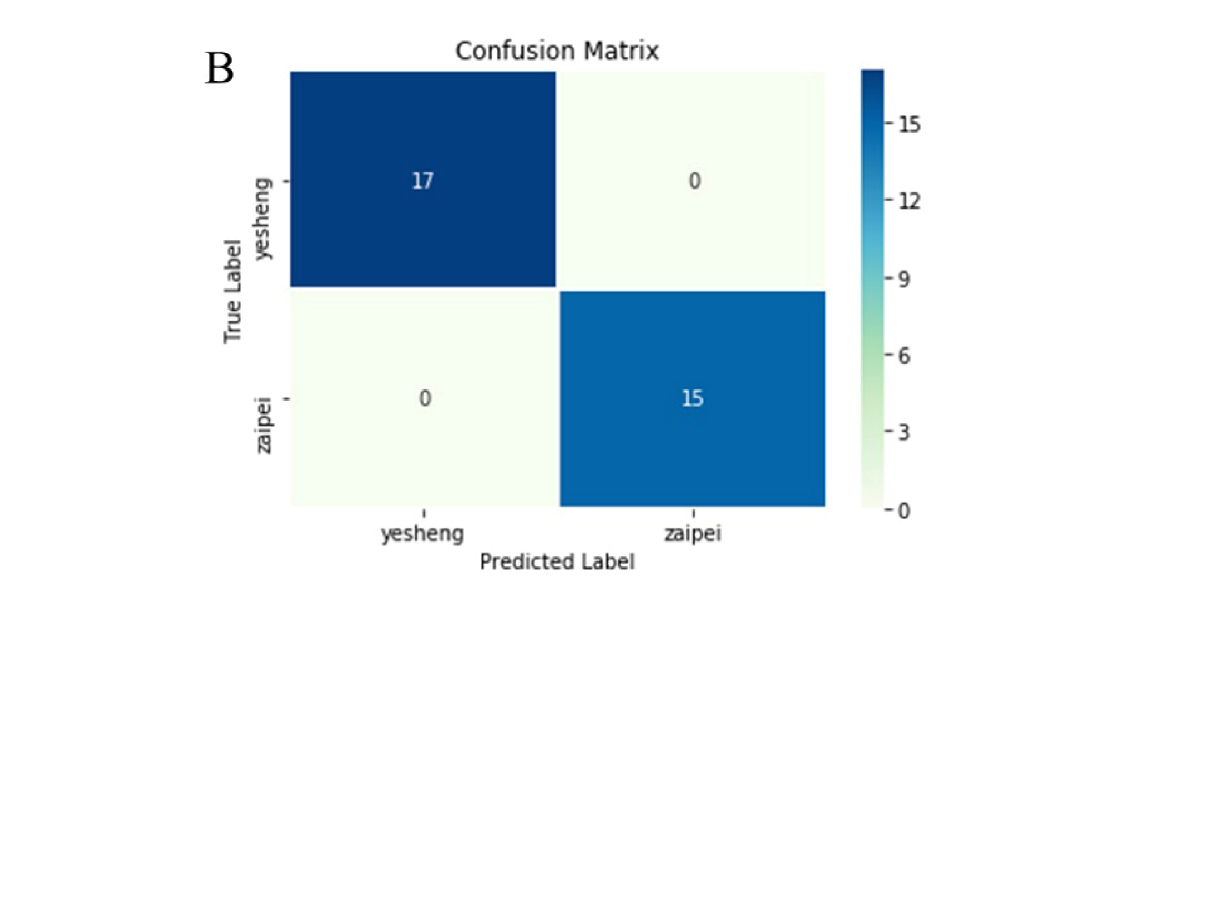


Figure S5. The confusion matrix of ResNet model based on synchronous 2DCOS spectra: (A) Poria and (B) Poriae Cutis.


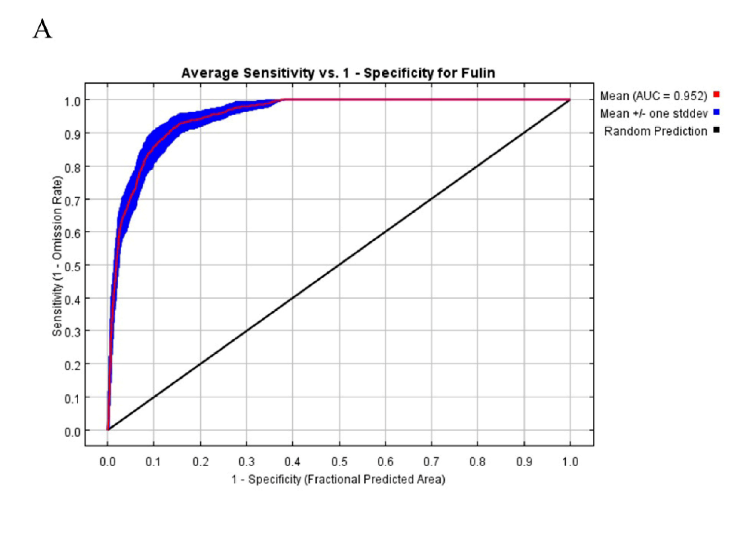

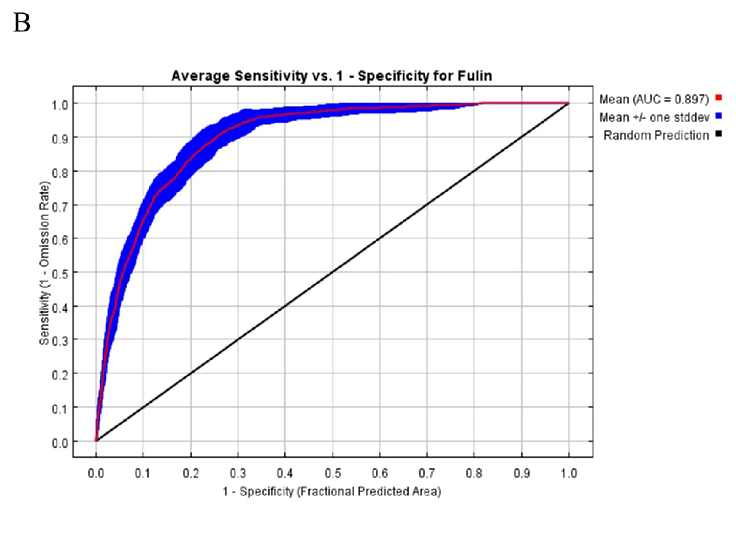


Figure S6. ROC curves of MaxEnt models for *W. cocos*: (A) climate and (B) soil.


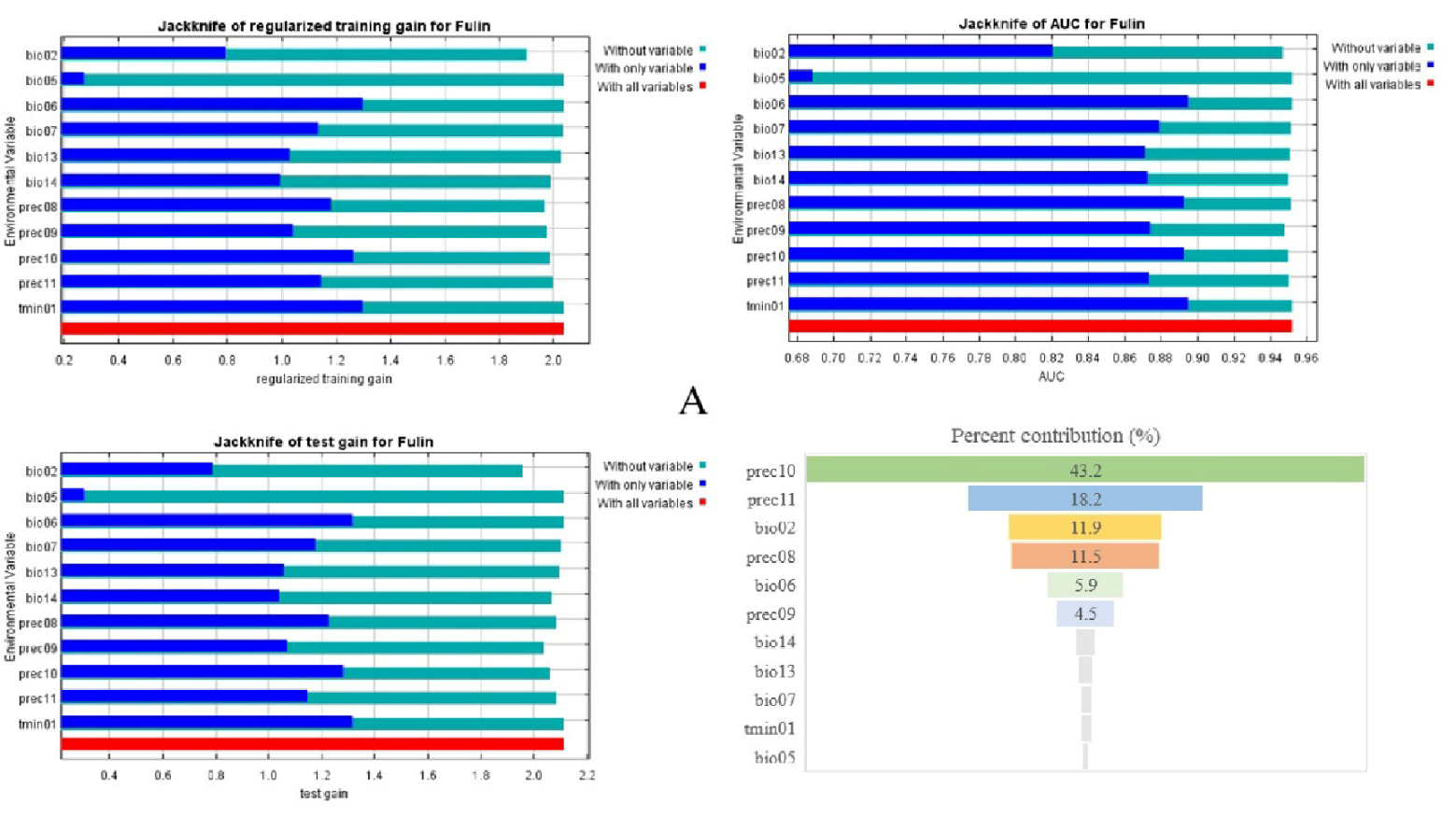


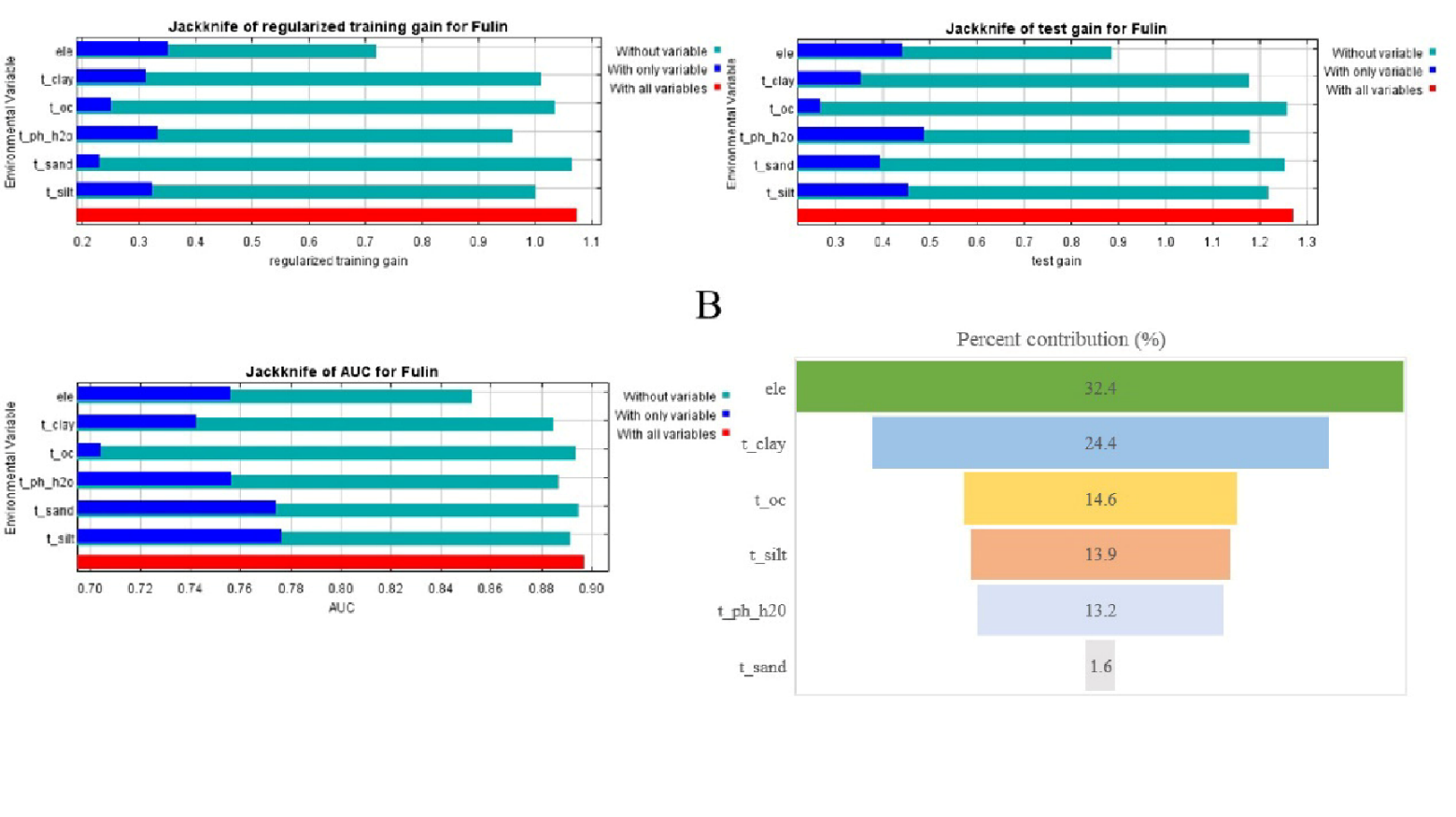


Figure S7. The results of the jackknife test and the environment variables’ percent contributions: (A) climate variables and (B) soil variables.


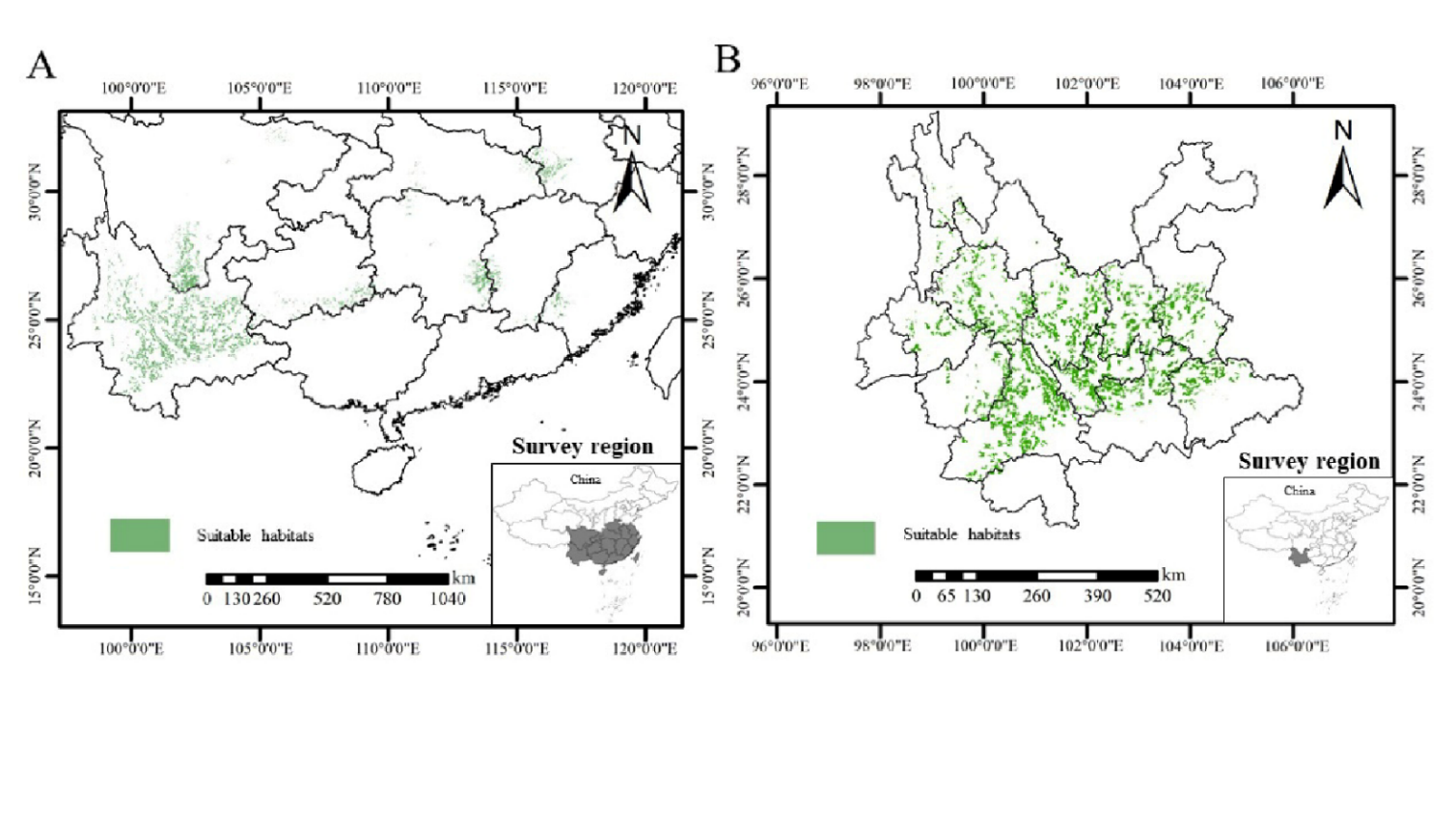


Figure S8. The suitable habitat of *W. cocos* distributed in (A) China and (B) Yunnan Province.

Table S1 The environmental variables information.

| Type | Code | Description | Unit |
| --- | --- | --- | --- |
| Topographic factor | Ele | Elevation | m |
| Climatic factors | Bio01 | Annual Mean Temperature | ℃ |
|  | Bio02 | Mean Diurnal Range | ℃ |
|  | Bio03 | Isothermality | / |
|  | Bio04 | Temperature Seasonality | / |
|  | Bio05 | Max Temperature of Warmest Month | ℃ |
|  | Bio06 | Min Temperature of Coldest Month | ℃ |
|  | Bio07 | Temperature Annual Range | ℃ |
|  | Bio08 | Mean Temperature of Wettest Quarter | ℃ |
|  | Bio09 | Mean Temperature of Driest Quarter | ℃ |
|  | Bio10 | Mean Temperature of Warmest Quarter | ℃ |
|  | Bio11 | Mean Temperature of Coldest Quarter | ℃ |
|  | Bio12 | Annual Precipitation | mm |
|  | Bio13 | Precipitation of Wettest Month | mm |
|  | Bio14 | Precipitation of Driest Month | mm |
|  | Bio15 | Precipitation Seasonality | / |
|  | Bio16 | Precipitation of Wettest Quarter | mm |
|  | Bio17 | Precipitation of Driest Quarter | mm |
|  | Bio18 | Precipitation of Warmest Quarter | mm |
|  | Bio19 | Precipitation of Coldest Quarter | mm |
|  | Prec (01-12) | Precipitation | mm |
|  | Tavg (01-12) | Average Temperature | ℃ |
|  | Sard (01-12) | Solar Radiation | kJ/m^2^/day^1^ |
|  | Tmax (01-12) | Max Temperature | ℃ |
|  | Tmin (01-12) | Min Temperature | ℃ |
|  | Aat 10 | ≥10℃ Active Accumulated Temperature | ℃ |
| Soil variables | T_Clay | Topsoil Clay Fraction | % weight |
|  | T_OC | Topsoil Organic Carbon | % weight |
|  | T_pH_H_2_O | Topsoil pH (H_2_O) | / |
|  | T_Sand | Topsoil Sand Fraction | % weight |
|  | T_Silt | Topsoil Silt Fraction | % weight |

Table S2 Environmental variables used to predict the potential geographic distribution of *M. cocos*.

| Code | Description | Unit |
| --- | --- | --- |
| Prec 08 | Precipitation in August | mm |
| Prec 09 | Precipitation in September | mm |
| Prec 10 | Precipitation in October | mm |
| Prec 11 | Precipitation in November | mm |
| Bio02 | Mean Diurnal Temperature Range | ℃ |
| Bio05 | Max Temperature of Warmest Month | ℃ |
| Bio06 | Min Temperature of Coldest Month | ℃ |
| Bio07 | Temperature Annual Range | ℃ |
| Bio13 | Precipitation of Wettest Month | mm |
| Bio14 | Precipitation of Driest Month | mm |
| Tmin 01 | Min Temperature in January | ℃ |
| Ele | Elevation | m |
| T_Clay | Topsoil Clay Fraction | % weight |
| T_OC | Topsoil Organic Carbon | % weight |
| T_pH_H_2_O | Topsoil pH (H_2_O) | / |
| T_Sand | Topsoil Sand Fraction | % weight |
| T_Silt | Topsoil Silt Fraction | % weight |
